# Chronic Unpredictable Mild Stress Promotes Atherosclerosis via HMGB1/TLR4-Mediated Downregulation of PPARγ/LXRα/ABCA1 in ApoE^-/-^ Mice

**DOI:** 10.3389/fphys.2019.00165

**Published:** 2019-03-01

**Authors:** Hong-Feng Gu, Na Li, Zhao-Qian Xu, Lu Hu, Hui Li, Rong-Jie Zhang, Ru-Meng Chen, Xi-Long Zheng, Ya-Ling Tang, Duan-Fang Liao

**Affiliations:** ^1^Department of Physiology and Institute of Neuroscience, University of South China, Hengyang, China; ^2^Division of Stem Cell Regulation and Application, State Key Laboratory of Chinese Medicine Powder and Medicine Innovation in Hunan, Hunan University of Chinese Medicine, Changsha, China

**Keywords:** chronic stress, atherosclerosis, inflammation, HMGB1, toll-like receptor 4, ABCA1

## Abstract

**Background:** Although our previous studies have confirmed that the activation of TLR4 is implicated in the development of atherosclerosis induced by chronic unpredicted mild stress (CUMS), the underling mechanism is largely unclear. Here, we hypothesized that CUMS accelerates atherosclerotic development through lowering PPARγ/LXRα-ABCA1 expression via HMGB1/TLR4 signaling.

**Methods:** In present study, CUMS atherosclerotic animal models were established with AopE^-/-^ mice, and CUMS Raw 264.7 macrophage models were mimicked by high corticosterone treatment, These models were treated with Ethyl pyruvate (EP, an inhibitor of HMGB1), TLR4 inhibitor TAK-242, and PPARγ agonist RSG (Rosiglitazone) to test our hypothesis, respectively.

**Results:** Our results indicated that the protein levels of HMGB1, TLR4, and pro-inflammatory cytokines including IL-1β, TNF-α were elevated with the development of atherosclerosis in CUMS mice, while the expressions of PPARγ, LXRα, and ABCA1 declined. Notably, HMGB1 inhibition by EP reversed CUMS-induced atherosclerotic development, pro-inflammatory cytokines upregulation, and PPARγ/LXRα-ABCA1 downregulation. The same trend was observed in the stressed mice treatment with TAK-242. Further experimental evidences indicated that EP, TAK-242, and RSG treatment notably corrected foam cell formation, HMGB1 release, and down-regulation of LXRα and ABCA1 in CUMS Raw 264.7 macrophage model.

**Conclusion:** These results indicate that CUMS exacerbates atherosclerosis is likely via HMGB1-mediated downregulation of PPARγ/LXRα-ABCA1 through TLR4. These data reveal a novel mechanism by which CUMS aggravates atherosclerosis and may offer a potential therapeutic target for this disease.

## Introduction

Accumulating evidence indicates that chronic stress, especially chronic psychological stress, is a critical risk factor for atherosclerotic diseases (Kaplan et al., 1983; Rozanski et al., 1999; Empana et al., 2005; Bagheri et al., 2016). The mechanisms by which chronic stress factors contribute to the development of atherosclerosis are always the research focus (Empana et al., 2005; Everson-Rose et al., 2014; Peplinski et al., 2018). Our previous studies and other reports demonstrate that chronic psychological stress promotes atherosclerosis in animal models by eliciting chronic inflammation in aortic wall (Gu et al., 2009, 2012; Bernberg et al., 2012). Although substantial studies confirm that inflammation plays a critical role in atherogenesis induced by chronic stress (Empana et al., 2005; Ranjit et al., 2007; Wirtz and von Kanel, 2017), the precise mechanisms underlying this atherosclerosis-related inflammation elicited by stress remain largely unknown.

Recently, several studies have showed that Toll-like receptor 4 (TLR4) is a novel link between chronic stress and inflammation (Tang et al., 2015; Yang et al., 2017). TLR4 is expressed in cells within vessel wall. Its activation triggers inflammatory cytokines (such as MCP-1, IL-1β, and TNF-α) production and release, which results in inflammatory cascade and thereby promotes atherosclerosis. Impressively, our previous studies revealed that TLR4-mediated inflammation is involved in the progression of atherogenesis induced by chronic unpredictable mild stress (CUMS) (Gu et al., 2009; Tang et al., 2015). However, the underlying mechanism atherosclerosis-related inflammation elicited by TLR4 activation in the context of this chronic stress is still unclear.

High-mobility group box 1 (HMGB1) can function as cytokine to mediate gene transcription or act as damage associated molecular pattern molecule to initiate and sustain a sterile inflammatory response (Scaffidi et al., 2002). Under conditions of cell stress and tissue damage, HMGB1 is released from dead cells and activated immune cells to extracellular space. These extracellular HMGB1 will target TLR4 or/and the receptor for advanced glycation end products (RAGE) to promote inflammation. Previous studies have demonstrated that HMGB1 is involved in several immune-related diseases (Hirata et al., 2013; Zhang et al., 2017). A recent report indicates that elevated HMGB1 contributes to neuroinflammatory response in depression mouse model induced by CUMS (Wang et al., 2018). Given that, we therefore ask whether and how HMGB1 regulates TLR4-mediated inflammation in atherosclerosis induced by CUMS.

It has been manifested that ATP-binding cassette transporter 1 (ABCA-1) exhibits anti-atherosclerotic roles via promoting cholesterol efflux and inhibiting inflammation (Ogura et al., 2011; Bi et al., 2015; Lake et al., 2017). Chawla et al. (2001) reported that ABCA-1 expression is regulated by peroxisome proliferator-activated receptor gamma (PPARγ)-nuclear receptor liver X receptor alpha (LXRα) pathway. A recent study showed that TLR4 activation decreases PPARγ and LXRα protein expressions in ox-LDL induced vascular smooth muscle cells (VSMCs), and the down-regulation of these two proteins is reversed by TLR4 deficiency (Cao et al., 2017). Furthermore, our previous study confirmed that TLR4 is activated in CUMS ApoE^-/-^ mice (Tang et al., 2015). Collectively, these studies suggest that CUMS-induced activation of TLR4 can decrease ABCA1 expression via suppressing the PPARγ/LXRα signaling in ApoE^-/-^ mice.

Based on above-mentioned data, we hypothesized that elevated HMGB1 accelerates CUMS-induced atherosclerosis by inhibiting PPARγ/LXRα-ABCA1 pathway via TLR4 activation. In this study, CUMS ApoE^-/-^ mice model with atherosclerosis was established, and HMGB1 inhibitor or TLR4 inhibitor was used to test its role in atherosclerosis under CUMS. Our results revealed that HMGB1 is a critical signal for TLR4-mediated the down-regulation of PPARγ/LXRα-ABCA1, which accelerates CUMS-induced atherosclerosis.

## Materials and Methods

### Materials

EP (Ethyl pyruvate) and Oil Red O were purchased from Sigma-Aldrich (St. Louis, MO, United States). TAK-242 and PPARγ agonist Rosiglitazone (RSG) were provided by MedChemExpress Company (Princeton, NJ, United States). Antibodies against HMGB1, TLR4, and IL-1β, were from Cell Signaling Biotechnology (Danvers, MA, United States). Antibody against CD68 were from Novus Biologicals (United States). Antibodies against PPARγ, LXRα, and ABCA1 were obtained from Abcam Company (Cambridge, United Kingdom). Rabbit Antibody against actin was provided by Proteintech Group, Inc. (Chicago, IL, United States). Corticosterone, IL-1β, and TNF-α ELISA kits were obtained from Westang Company (Shanghai, China). HMGB1 parameter assay kits were purchased from Cusabio Biotech, Co., Ltd. (Wuhan, China).

### Animals

Sixty male ApoE^-/-^ mice (5 weeks old, 17.0 ± 0.5 g weight) were purchased from Model Animal Research Center of Nanjing University, Nanjing, China. The animals were housed 5 per cage (8 cm × 13.5 cm × 8.1 cm) and maintained in humidity and temperature-controlled conditions with a 12-h dark/light cycle (lights on at 7:00 AM), and were accessed to food and water freely. All the mice were given 1 week to adapt the new environment prior to experiments. The animals used in this study were approved by the Animal Experimentation Ethics Committee of the University of South China (Permit Number: XYXK2015-0001). All experimental procedures were strictly carried out in accordance with the guidelines published by the “China Council on Animal Care.”

### Experimental Design and Drug Treatments

After adaption for 1 week, the mice were randomly divided into the following four groups (*n* = 15 per group): control group, CUMS group, CUMS + EP (an inhibitor of HMGB1) group, and CUMS + TAK-242 (an inhibitor of TLR4) group. The animals in CUMS groups were exposed to unpredicted chronic mild stressors for 16 weeks. Vehicle (PBS) administrations or drug treatments were given from the beginning of CUMS exposure to the end of CUMS. EP (at a dose of 50 mg/kg, once daily) and TAK-242 (at a dose of 0.3 mg/kg, twice a week) were given by intraperitoneal injection for consecutive 16 weeks respectively. The doses of these two inhibitors were chosen based on previous reports (Ni et al., 2013; Xiao et al., 2016). All the mice were fed a normal chow diet.

### Chronic Unpredictable Mild Stress Protocol

Chronic unpredicted mild stress procedures were carried out as described in our previous studies (Gu et al., 2009; Tang et al., 2015). To prevent ApoE^-/-^ mice from adapting to CUMS, the animals were suffered the following stressors in a random order over each week: two 12-h sessions of overnight illumination; three 7-h sessions of 45 degrees cage tile; a 12-h session empty bottles stimulation; exposure to rat odor (keeping the experimental mice into cages in which rats had been held) for 1 h, twice a week; two 2-h sessions in an immobilization stress tube. Food consumption was measured by monitoring the food intake daily. The control mice were kept in a separate room and did not contact with the stressed ones. After 16 weeks CUMS, body weight, serum corticosterone (CORT) concentration, and sucrose preference were evaluated.

### Evaluation of CORT Levels in Serum

After overnight fasting at the final day of the CUMS protocol, the animals were anesthetized with 3% pentobarbital sodium solution (intraperitoneal injection). Then blood samples were collected by heart puncture. The serum corticosterone contents were measured by using commercially available reagent kits.

### Atherosclerotic Lesion Analysis

Atherosclerotic lesions within aortic sinus was analyzed by Oil Red O staining as previously described. In brief, the mice were perfused with 4% paraformaldehyde-sucrose solution for 30 min after being euthanized with 3% pentobarbital sodium solution. The aortic root was harvested. To evaluate atherosclerotic lesions in aortic sinus, aortic roots were fixed in 4% paraformaldehyde solution at 4°C overnight, and then embedded in OCT compound for cryosectioning. Serial cryostat sections 8 μm thick were harvested on slides for atherosclerotic lesion analysis by Oil Red O staining. The cross-sections were stained with Oil Red O and counterstained with hematoxylin. Atherosclerotic lesions sizes were analyzed (IMAGEPRO PLUS 6.3) by two blinded observers to the study protocol. For each animal, 10 serial cross-sections were determined. Results were presented as the percent of the whole aortic surface area of the root.

### Immunohistologic and Immunofluorescence Analysis of Atherosclerotic Lesions

Frozen-sections of aortic sinus were performed with hemotoxylin and eosin and Masson’s trichrome staining to evaluate necrotic areas and collagen content of the lesions, respectively. CD68 immunofluorescence staining by using anti-CD68 mAb (1: 500) was performed to detect macrophage content in atherosclerotic lesion. Rat IgG2b or mouse IgG2a was used as negative control for immunofluorescence staining. The samples were counterstained with DAPI for fluorescence photography. To avoid non-specific antibody binding, a blocking procedure was performed by pre-incubation with FcBlock and 1% goat serum prior to staining with primary mAbs. Percentage of positive staining area of all serial sections in each group was elevated by image analysis software (IMAGEPRO PLUS 6.3).

### Cell Culture and Treatment

Raw 264.7 macrophage cells was purchased from Chinese Academy of Sciences (Shanghai, China), and cultured in DMEM medium (Sigma) supplemented with 10% FBS at 37°C in atmosphere containing 5% CO_2_. Raw 264.7 macrophages were treated with 20 μg/mL of ox-LDL with or without HMGB1 inhibitor EP, TLR4 inhibitor TAK-242, and PPARγ agonist RSG in presence or absence of 5 μM of CORT for 36 h.

After 36 h treatment, the cultured cells were harvested for assessing foam cell formation by Oil Red O staining, and for TLR4, PPARγ, LXRα, and ABCA1 expression by Western blot Analysis. The release of HMGB1 in cell culture supernatant was detected by Enzyme-linked immunosorbent assay (ELISA).

### Western Blot Analysis

Mouse aortas were harvested for protein extraction and detection of HMGB1, TLR4, IL-1β, PPARγ, LXRα and ABCA1 as described previously. Briefly, sample protein concentrations were quantified by using a BCA Protein Assay Kit (Beyotime, Shanghai, China). Equal amounts of the boiled proteins (20 μg per lane) from each sample were separated by an 8% sodium dodecyl sulfate-polyacrylamide gel electrophoresis and then wet electro-transferred to a polyvinylidene fluoride (PVDF) membrane (Millipore). After blocking with 5% bovine serum albumin at room temperature for 1 h, the membranes were first incubated with primary antibody against actin (1:2000), HMGB1 (1:1000), TLR4 (1:1000), IL-1β (1:1000), PPARγ (1:1000), LXRα (1:1000), and ABCA1 (1:1000) at 4°C overnight, respectively. Then, the membranes were rinsed with TBST and subsequently incubated with horseradish peroxide conjugated secondary antibodies (1:3000) for 1 h at room temperature. Finally, immunoreactivity was developed with ECL fluorescent detection reagent. Densitometry analysis of the blots was carried out by using the NIH Image J software. Blots against β-actin served as control.

### Enzyme-Linked Immunosorbent Assay (ELISA)

Protein levels of HMGB1, TNF-α, and IL-1β in serum were determined by ELISA kits following the manufacturer’s instructions. In brief, serum was added to 96-well plates (100 μL per well) in duplicate. Minimum detection limit of HMGB1, TNF-α, and IL-1β was 3.9, 4, and 8 pg/mL respectively.

### Statistical Analysis

Results were analyzed by GraphPad Prism 7 statistical software. All data were expressed as mean ± SEM Student’s *t*-test (comparisons between two groups) or one-way analysis of variance (comparisons among multiple groups) was used to perform statistical analysis. Statistical significance was set at *P* < 0.05.

## Results

### EP or TAK-242 Treatment Had No Significant Effect on Serum CORT Contents in CUMS ApoE^-/-^ Mice, but Obviously Lowered Serum HMGB1 Levels in Them

**FIGURE 1:**
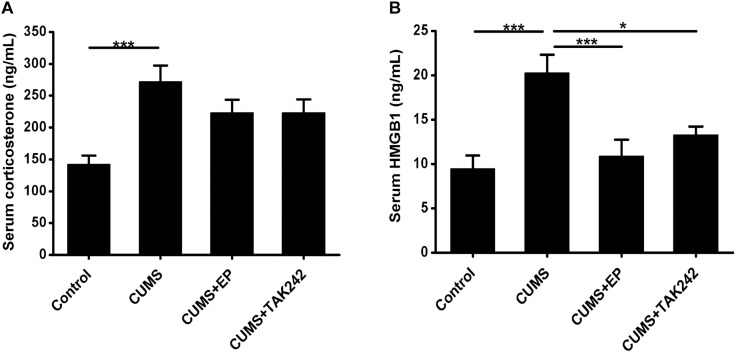
Effect of EP and TAK-242 treatment on serum corticosterone concentrations and HMGB1 protein levels in CUMS ApoE^-/-^ mice. ApoE^-/-^ mice were injected with vehicle (PBS), EP (50 mg/kg, once daily), or TAK-242 (0.3 mg/kg, twice a week) for consecutive 16 weeks by intraperitoneal injection 30 min prior to CUMS. Over a 16-week period treatment, blood samples were collected for assessment of corticosterone and HMGB1 contents. **(A)** Serum corticosterone profiles. Corticosterone levels in serum were determined by using commercially available reagent kits. **(B)** HMGB1 protein levels in serum were measured by ELISA. Data were expressed as mean ± SEM (*n* = 15). Statistical analysis was performed by Student’s *t*-test or one-way analysis of variance. ^∗^*P* < 0.05, ^∗∗∗^*P* < 0.001.

To confirm whether CUMS paradigms used in this study could induce sufficient systemic stress, serum CORT levels in ApoE^-/-^ mice were measured. As shown in Figure 1A, the serum CORT contents in the stressed mice were much higher than those in the normal control ones, indicating that CUMS exposure triggered an effective systemic stress response in the mice. Interestingly, the serum CORT levels both in CUMS mice treatment with EP and TAK-242 were not significantly declined as compared to the mice treatment with CUMS alone.

To determine whether CUMS could increase the release of HMGB1, its protein concentrations in serum were measured by ELISA assay. As shown in Figure 1B, HMGB1 protein levels in CUMS mice were significantly elevated as compared to those in the normal control mice. As expected, treatment with EP or TAK-242 almost abolished the elevating trend of serum HMGB1 in CUMS ApoE^-/-^ mice. Based on this, we tended to further investigate the effect of HMGB1/TLR4 signaling on the development of atherosclerosis induced by CUMS.

### HMGB1 or TLR4 Inhibition Attenuates Atherosclerotic Lesions in CUMS ApoE^-/-^ Mice

**FIGURE 2:**
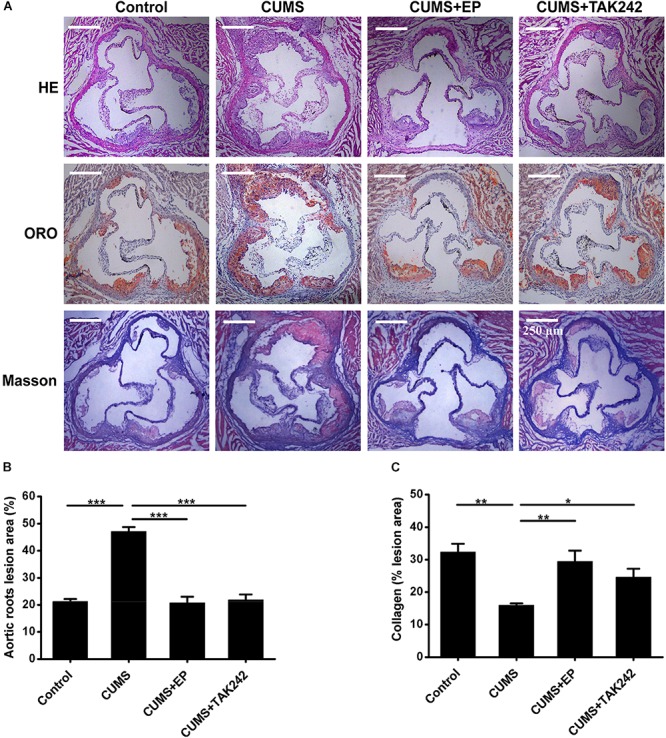
Effect of EP and TAK-242 treatment on atherosclerotic lesions in CUMS ApoE^-/-^ mice. Over a 16-week period treatment, the aortic root was harvested for analysis of atherosclerotic lesions. **(A)** Representative images of H&E, ORO (Oil Red O, ORO), and Masson staining of aortic sinuses. **(B)** Quantification of atherosclerotic plaque areas of aortic sinuses revealed that EP or TAK-242 treatment significantly decreased atherosclerotic lesions induced by CUMS (*n* = 15). **(C)** Quantification of collagen fibers content of aortic sinuses (*n* = 15). Data were expressed as mean ± SEM. Statistical analysis was performed by Student’s *t*-test or one-way analysis of variance. ^∗^*P* < 0.01, ^∗∗^*P* < 0.01, ^∗∗∗^*P* < 0.001.

To confirm the potential role and underlying mechanism of HMGB1 in CUMS-induced atherosclerosis, CUMS ApoE^-/-^ mice were treated with HMGB1 inhibitor EP and TLR4 inhibitor TAK-242, respectively. Atherosclerotic lesions in the aortic sinus of the mice were valuated by Oil Red O staining. As shown in Figure 2A,B, CUMS ApoE^-/-^ mice developed more atherosclerotic lesions in aortic sinus than those in normal control ones, indicting a profound aggravation of atherosclerosis by CUMS. Also, the plaque phenotype in CUMS mice was obviously altered as collagen content decreased in the atherosclerotic lesions (Figure 2C). Expectedly, EP or TAK-242 treatment not only significantly attenuated atherosclerotic lesions of CUMS ApoE^-/-^ mice, but also profoundly increased atherosclerotic collagen content. Those results revealed that inhibition of HMGB1/TLR4 can prevent the development of atherosclerosis induced by CUMS.

### HMGB1 or TLR4 Inhibition Decreases the Expressions of HMGB1, TLR4, and IL-1β Proteins in Aorta of ApoE^-/-^ Mice Under CUMS

**FIGURE 3:**
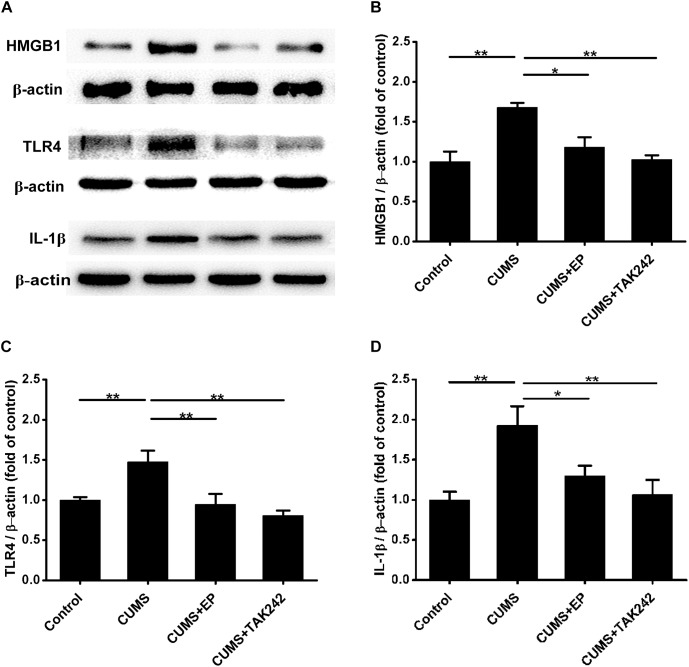
Effect of EP and TAK-242 treatment on HMGB1, TLR4, and IL-1β protein expressions in aorta of CUMS ApoE^-/-^ mice. Over a 16-week period treatment, entire aorta from the aortic root to the iliac bifurcation was harvested for Western blot analysis. **(A)** Representative Western blots of HMGB1 (29 kDa), TLR4 (95 kDa), and IL-1β (17 kDa). **(B–D)** Quantification of HMGB1, TLR4, and IL-1β protein levels by the NIH Image J software revealed that EP and TAK-242 treatment significantly lowered those three proteins expression in the aorta of the CUMS apoE^-/-^ mice. Data were expressed as mean ± SEM (*n* = 5). Statistical analysis was performed by Student’s *t*-test or one-way analysis of variance. ^∗^*P* < 0.05, ^∗∗^*P* < 0.01.

Compelling evidences indicate that HMGB1, TLR4, and IL-1β are implicated in the progression of atherosclerosis (Kirii et al., 2003; Kalinina et al., 2004; Michelsen et al., 2004). We next explored the influence of EP and TAK 242 treatments on the expressions of those proteins in aorta of the CUMS ApoE^-/-^ mice by Western blotting assay. As shown in Figure 3, the expressions of HMGB1, TLR4, and IL-1β were profoundly increased in aorta of CUMS group as compared to those in the control group, respectively. However, EP treatment dramatically decreased the above three proteins levels in the aorta of the CUMS ApoE^-/-^ mice, respectively. Also, the same trend was observed in aorta of the CUMS + TAK-242 mice. These results demonstrated that the activation of HMGB1/TLR4 plays a crucial role in atherogenesis of CUMS ApoE^-/-^ mice.

### HMGB1 or TLR4 Inhibition Reduces Accumulation of CD68+ Macrophages in Aorta of ApoE^-/-^ Mice Under CUMS

**FIGURE 4:**
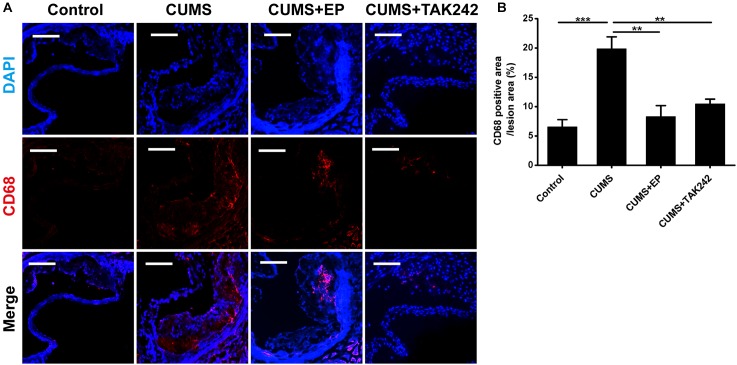
Effect of EP and TAK-242 treatment on CD68 protein expressions in aortic sinus of CUMS ApoE^-/-^ mice. Over a 16-week period treatment, entire aorta from the aortic root to the iliac bifurcation was harvested, and then frozen-sections of aortic sinus were performed for immunofluorescent staining of CD68. **(A)** Representative images of CD68 expression in aortic sinus. **(B)** Quantification of CD68 positive areas in aortic sinus lesions revealed that inhibition of HMGB1 and TLR4 caused in a significant decrease in infiltrating macrophages induced by CUMS. CD68-positive cells seem in red, and nuclei in blue. Data were expressed as mean ± SEM (*n* = 5). Statistical analysis was performed by Student’s *t*-test or one-way analysis of variance. ^∗∗^*P* < 0.01, ^∗∗∗^*P* < 0.01.

Considering the activation of HMGB1 and TLR4 plays key roles in atherosclerotic progression and plaque stability, we therefore further investigated the effects of EP and TAK-242 on the pathological characteristics of plaque in ApoE^-/-^ mice exposed to CUMS. As shown in Figure 4A,B, CD68 immunofluorescence staining-positive areas in the plaque of CUMS group were much larger those in the control group. As expected, EP or TAK-242 treatment dramatically decreased CD68 immunofluorescence staining-positive areas in the atherosclerotic lesions of ApoE^-/-^ mice under CUMS. Those results revealed that CUMS-induced an increase of HMGB1 exacerbated macrophage aggregation in atherosclerotic lesions of CUMS ApoE^-/-^ mice via TLR4 signaling.

### HMGB1 or TLR4 Inhibition Significantly Lowers Serum IL-1β, and TNF-α Protein Levels in ApoE^-/-^ Mice Under CUMS

**FIGURE 5:**
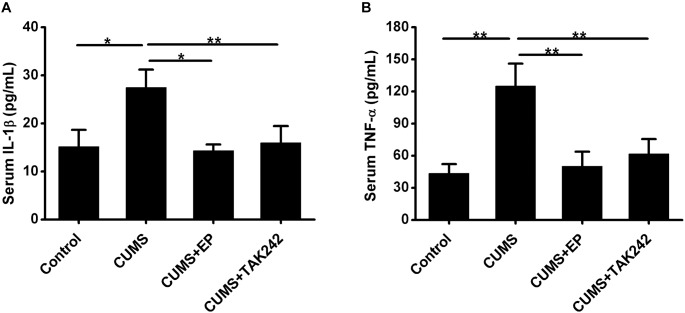
Effect of EP and TAK-242 treatment on serum IL-1β and TNF-α protein levels in CUMS ApoE^-/-^ mice. ApoE^-/-^ mice were injected with vehicle (PBS), EP (50 mg/kg, once daily), or TAK-242 (0.3 mg/kg, twice a week) for consecutive 16 weeks by intraperitoneal injection 30 min prior to CUMS. Over a 16-week period treatment, blood samples were collected for detection of serum IL-1β and TNF-α protein levels by ELISA. **(A,B)** Quantification of expression levels by ELISA indicated increases in serum IL-1β and TNF-α protein contents in CUMS apoE^-/-^ mice, whereas HMGB1 inhibition by EP or TLR4 inhibition by TAK-242 abolished CUMS-induced increases in serum IL-1β and TNF-α levels. Data were expressed as mean ± SEM (*n* = 15). Statistical analysis was performed by Student’s *t*-test or one-way analysis of variance. ^∗^*P* < 0.05, ^∗∗^*P* < 0.01.

The activation of HMGB1/TLR4 signaling plays vital roles in CUMS-induced systemic inflammation. We next asked whether EP or TAK-242 treatment ameliorated atherosclerotic lesions in CUMS mice might be associated with the reduced systemic inflammation cytokine levels. In present study, ELISA analysis was used to measure serum IL-1β and TNF-α protein levels in ApoE^-/-^ mice. As shown in Figure 5, compared with the CUMS group, the concentrations of serum IL-1β and TNF-α protein were much higher than those in control group, respectively. However, those serum protein levels potently decreased both in EP and TAK-242 treatment group as compared to those in the CUMS group. These results manifested that the elevated HMGB1 protein levels resulting from CUMS contributed to the systemic inflammation in ApoE^-/-^ mice via activation of TLR4.

### Inhibiting HMGB1 or TLR4 Reverses CUMS-Induced the Down-Regulation of PPARγ, LXRα, and ABCA1 Expressions in Aorta of ApoE^-/-^ Mice

**FIGURE 6:**
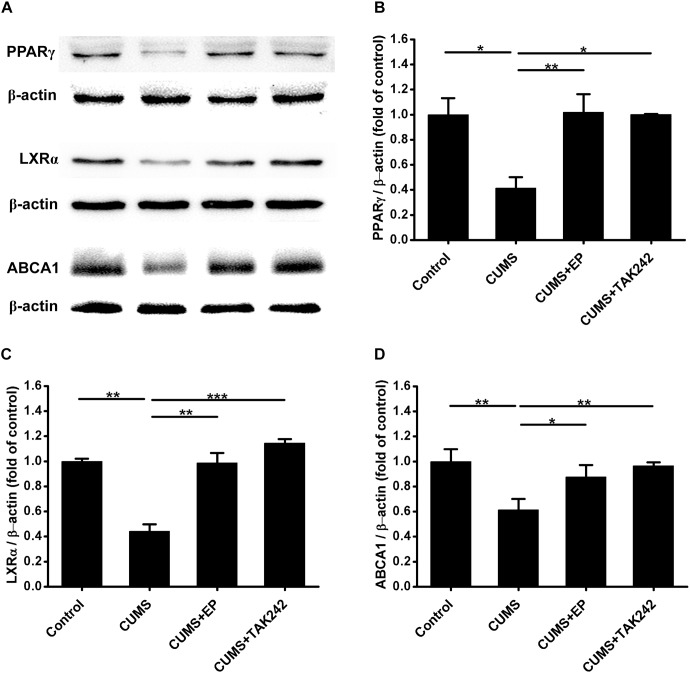
Effect of EP and TAK-242 treatment on the expressions PPARγ, LXRα, and ABCA1 protein levels in aorta of CUMS ApoE^-/-^ mice. Over a 16-week period treatment, entire aorta from the aortic root to the iliac bifurcation was harvested for Western blot analysis. **(A)** Representative Western blots of PPARγ (57 kDa), LXRα (50 kDa), and ABCA1 (254 kDa). **(B–D)** Quantification of PPARγ, LXRα, and ABCA1 protein levels by the NIH Image J software revealed drown-regulation of PPARγ, LXRα, and ABCA1 proteins in aorta of CUMS ApoE^-/-^ mice, whereas HMGB1 inhibition by EP or TLR4 inhibition by TAK-242 significantly reversed CUMS-induced the down-regulation of these three proteins in aorta. Data were expressed as mean ± SEM (*n* = 5). Statistical analysis was performed by Student’s *t*-test or one-way analysis of variance. ^∗^*P* < 0.05, ^∗∗^*P* < 0.01, ^∗∗∗^*P* < 0.001.

Considerable evidence indicates that ABCA1 plays a crucial role in atherosclerotic development, and the expression of this ATP binding cassette transporter is regulated by PPARγ/LXRα signaling. Knowing that TLR4 activation can inhibit the expression of PPARγ, we next further explore the mechanism by which CUMS-enhanced HMGB1/TLR4 signaling contributed to atherosclerotic lesion development in ApoE^-/-^ mice. The protein levels of PPARγ, LXRα, and ABCA1 in aorta of ApoE^-/-^ mice were measured by Western blot analysis. As shown in Figure 6A–D, PPARγ, LXRα, and ABCA1 protein expressions were significantly decreased in CUMS group when compared with the control group. However, EP blockage of HMGB1 or TAK-242 inhibitor of TLR4 treatment remarkably reversed CUMS-induced the down-regulation of these three protein levels in the aorta of ApoE^-/-^ mice. These results implied that CUMS-enhanced HMGB1 protein levels drown-regulates PPARγ, LXRα, and ABCA1 expressions in the aorta of ApoE^-/-^ mice via TLR4.

### EP, TAK-242, and PPARγ Agonist RSG Treatment Abrogates Raw 264.7 Macrophage-Derived Foam Cell Formation Induced by High Level CORT

**FIGURE 7:**
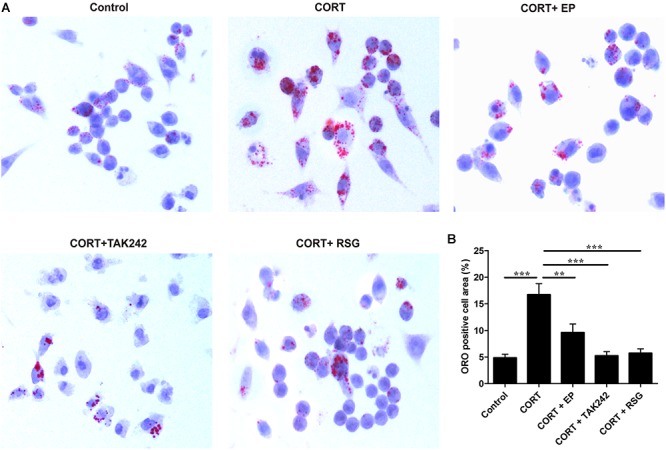
Effect of EP, TAK-242, and RSG treatment on high level CORT-induced Raw 264.7 macrophage foam cell formation. Raw 264.7 macrophage cells were cultured ox-LDL (20 μg/mL) with or without EP (5 mM), TAK-242 (1 μM), and RSG (10 μM), in present or absence of 5 μM of CORT for 36 h. **(A)** Macrophages were fixed and stained with Oil Red O to evaluate foam cell formation (original magnification: 40×). **(B)** Quantification of Oil Red O (ORO) staining positive areas. Data were expressed as mean ± SEM (*n* = 3). ^∗∗^*P* < 0.01, ^∗∗∗^*P* < 0.001.

Atherosclerosis is developed progressively with the deposition of foam cells within vessel walls. To further confirm whether the activation of HMGB1/TLR4 promotes CUMS-induced atherogenesis via inhibiting PPARγ/LXRα-ABCA1 signaling, Raw 264.7 macrophages were treated with EP, TAK-242 or RSG in present with CORT for 36 h to mimic CUMS *in vitro*, and then the intracellular lipid accumulation, and the expressions of PPARγ/LXRα/ABCA1 were measured. As shown in Figure 7A,B, lipid accumulation in CORT treatment group was increased significantly as compared with control group, indicating that high concentration of CORT promotes foam cell formation. As expected, the above effects of CORT on foam cell formation were obviously diminished by EP, TAK-242 and RSG, respectively.

### EP, TAK-242, and PPARγ Agonist RSG Treatments Abrogate Downregulation of PPARγ, LXRα, and ABCA1 Induced by High Level CORT in Raw 264.7 Cell

**FIGURE 8:**
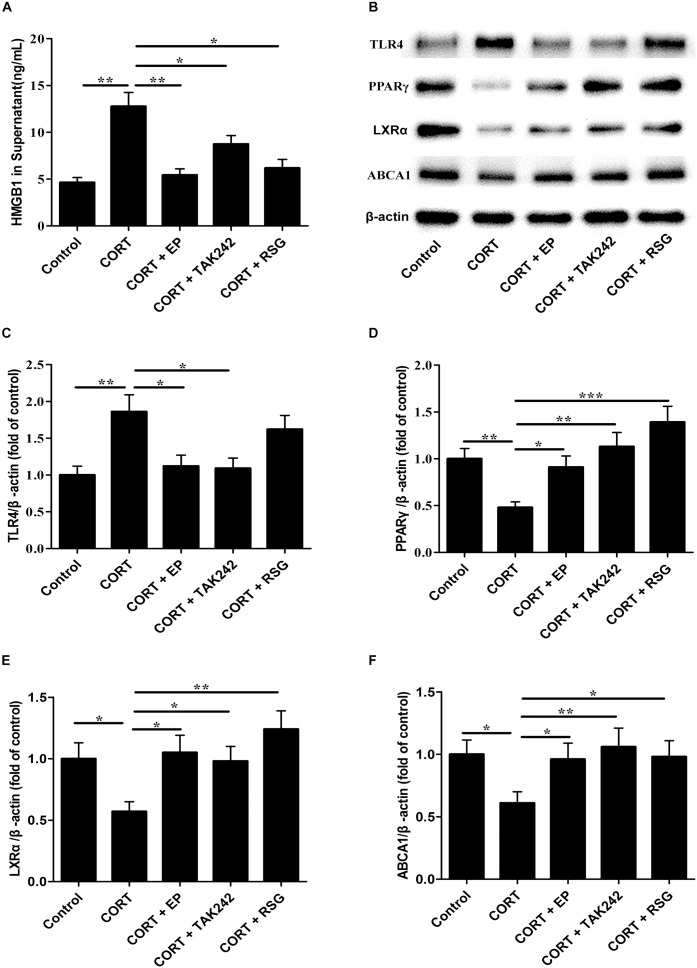
Effect of EP, TAK-242, and RSG treatment on high level CORT-induced HMGB1 release, and TLR4, PPARγ, LXRα, and ABCA1 protein expression in Raw 264.7 macrophage. Raw 264.7 macrophage cells were cultured oxLDL (20 μg/mL) with or without EP (5 mM), TAK-242 (1 μM), and RSG (10 μM), in present or absence of 5 μM of CORT for 36 h. **(A)** HMGB1 release of HMGB1 in supernatant was analyzed by ELISA. **(B)** Representative Western blots of TLR4 (95 kDa), PPARγ (57 kDa), LXRα (50 kDa), and ABCA1 (254 kDa). **(C–F)** Quantification of TLR4, PPARγ, LXRα, and ABCA1 protein levels by the NIH Image J software in the cultured macrophage cells. Data were expressed as mean ± SEM (*n* = 3). ^∗^*P* < 0.01, ^∗∗^*P* < 0.01, ^∗∗∗^*P* < 0.001.

Finally, the HMGB1 levels in cell culture supernatant, and the protein levels of TLR4, PPARγ, LXRα, and ABCA1 in Raw 264.7 cells were evaluated. As shown in Figure 8A, HMGB1 contents in cell culture supernatant were significantly elevated in CORT group as compared with that in control group, suggesting that CUMS induces macrophage activation and HMGB1 release. As indicated in Figure 8B–F, TLR4 expression in CORT group was significantly enhanced in parallel with HMGB1 release, while the expressions of PPARγ, LXRα, and ABCA1 were notably declined. However, the inhibiting effects of CORT on PPARγ, LXRα, and ABCA1 expression were canceled by EP, TAK-242, and RSG treatment. These results suggested that CUMS lowered PPARγ, LXRα, and ABCA1 expression via activation of HMGB1/TLR4.

## Discussion

In the present work, we offer new insights into the molecular mechanisms underlying the development of atherosclerosis induced by CUMS in ApoE^-/-^ mice. The major findings were as follows. (1) Along with CUMS-induced the elevated HMGB1 protein levels both in serum and aorta, TLR4 and its downstream pro-inflammatory cytokines increased in ApoE^-/-^ mice, while CUMS-induced those effects almost reversed by HMGB1 inhibitor EP. (2) HMGB1 or TLR4 blockage not only significantly decreased aortic sinus atherosclerosis lesions, but also increased plaque stability. (3) Importantly, inhibition of HMGB1 or TLR4 almost reversed CUMS-induced the inhibition of PPARγ, LXRα, and ABCA1 expressions in aorta of ApoE^-/-^ mice. These findings indicate that the activation of TLR4 by CUMS-induced HMGB1 promotes atherosclerosis in ApoE^-/-^ mice maybe via inhibiting PPARγ/LXRα/ABCA1 signaling.

Hypothalamic–pituitary–adrenal (HPA) axis dysfunction has been confirmed to be a critical factor in stress-related diseases, such as depressive disorders and cardiovascular diseases (Harkness and Monroe, 2006; Lamers et al., 2013). In this study, HPA axis of the CUMS mice exhibited hyperactivity, as indicated by significantly elevated serum corticosterone levels. This finding is in agreement with other studies. Interestingly, there were no significant changes in serum corticosterone contents between CUMS and EP, and TAK-242 treatment CUMS mice, suggesting that inhibiting HMGB1 release and TLR4 activation has no significant influence on HPA hyperactivity of the CUMS mice.

It has been well-established that HMGB1 is a key mediator in sterile inflammation, and that it highly expressed in atherosclerotic plaques (Inoue et al., 2007; Cheng et al., 2017). Once HMGB1 is released from nucleus to extracellular by the damaged cells or activated macrophage cells, it can initiate and sustain inflammation. Wang et al. found that HMGB1 levels were obviously elevated in the hippocampus and serum of the mice with depression induced by CUMS (Wang et al., 2018). In this study, we demonstrated that CUMS significantly increased atherosclerosis lesions as well as HMGB1 levels both in serum and aortas of ApoE^-/-^ mice. Furthermore, not only the pro-inflammatory cytokine levels such as IL-1β, and TNF-α, but also the CD68 positive cells in aorta of CUMS mice were elevated in parallel with HMGB1. Impressively, EP treatment almost reversed these alterations in ApoE^-/-^ mice under CUMS, indicating that CUMS induces inflammatory response in vessel wall by increasing HMGB1 production and release, thereby promoting atherosclerotic development. However, the mechanism by which HMGB1 contributes to atherogenesis in ApoE^-/-^ mice under CUMS is still unclear.

TLR4 is the other major receptor for HMGB1 except RAGE to trigger inflammation (Andersson and Tracey, 2011). In previous studies, we found that CUMS significantly upregulates TLR4 expression and promotes nuclear factor-kappa B (NF-κB) activation in aorta of ApoE^-/-^ mice (Gu et al., 2009; Wang et al., 2018). However, the upstream signal that leads to the activation of TLR4 signaling remains further exploration. In this work, our results manifested that concurrent with the rise in HMGB1 level were the increases in TLR4 and inflammatory cytokine expressions in the aorta of CUMS ApoE^-/-^ mice. In contrast, the CUMS mice treatment with EP, the specific inhibitor of HMGB1 releasing, exhibited decreases in HMGB1 and TLR4 protein concentrations, and reductions in the downstream inflammatory cytokine IL-1β and TNF-α expression. Moreover, the present work indicated that the TLR4 specific inhibitor TAK-242 not only attenuated CUMS-induced atherosclerotic development, but it also remarkably lowered the inflammation associated with HMGB1 signaling. Although compelling studies show that HMGB1 and TLR4 play pivotal roles in the pathogenesis of cardiovascular diseases and inflammatory response, but there was no literature about HMGB1/TLR4 signaling contributes to atherogenesis induced by CUMS. Therefore, our study provided a novel mechanism that CUMS-induced HMGB1 promotes atherosclerosis via TLR4.

The major role of ABCA1 is to remove intracellular excess lipids and to inhibit inflammation (Yin et al., 2010; Bi et al., 2015; Ren et al., 2018). Studies have indicated that ABCA1 expression is decreased in atherosclerotic lesion, and this decrease is associated with the enhanced inflammatory response and atherosclerotic progression (Su et al., 2005; Mukhamedova et al., 2011). However, the effects of HMGB1/TLR4 activation induced by CUMS on ABCA1-mediated inflammatory response and atherosclerotic development are largely unknown. Hence, in present study, we further explored whether CUMS induced HMGB1/TLR4 signaling might influence the expression of ABCA1. Our results demonstrated that CUMS exposure significantly elevated the expressions of HMGB1 and TLR4 proteins in aorta of ApoE^-/-^ mice, while lowered the levels of ABCA1 protein in aorta. In contrast, treatment with EP profoundly decreased TLR4 expression and increased ABCA1 expression in CUMS mice. Furthermore, TLR4 inhibitor TAK-242 administration notably enhanced the expression of ABCA1 in aorta of ApoE^-/-^ mice under CUMS. Therefore, our present work was the first to reveal that enhancing HMGB1/TLR4 signaling induced by CUMS declined the expression of ABCA1 in aorta, leading to the development of atherosclerosis as confirmed by aortic sinus Oil Red O staining.

Several studies have confirmed that PPARγ plays a critical role in promoting ABCA1 expression and cholesterol efflux through LXRα (Hou et al., 2007; Ozasa et al., 2011; Lin et al., 2017). Therefore, we further investigated the expressions of PPARγ and LXRα in aorta of CUMS ApoE^-/-^ mice. Our results indicated that CUMS significantly decreased PPARγ and LXRα protein levels in aorta. These findings suggest that PPARγ-LXRα-ABCA1 pathway is implicated in CUMS-induced atherosclerosis in ApoE^-/-^ mice. While the mechanism of the reduced PPARγ-LXRα-ABCA1 expression in CUMS ApoE^-/-^ mice is still unknown, we proposed that it could be via HMGB1/TLR4 signaling. In this study, we found that EP or TAK-242 treatment reversed the downregulated expression of PPARγ and LXRα proteins in aorta of CUMS ApoE^-/-^ mice. Also CUMS cell model mimicked by high level CORT administration demonstrated that RSG treatment profoundly corrected CORT-induced foam cell formation and drown-regulation of LXRα and ABCA1. It has been reported that the activation of TLR4 declines PPARγ expression (Cao et al., 2017). Our previous studies showed that CUMS results in the activation of TLR4 (Gu et al., 2009; Tang et al., 2015). Taken together, these results indicated that inhibiting PPARγ-LXRα expression may be a potential mechanism that contributes to HMGB1/TLR4-mediated ABCA1 downregulation under CUMS.

## Conclusion

Our study demonstrates that HMGB1 promotes CUMS-induced atherosclerosis via activating TLR4 signaling, which drownregulates the expression of PPARγ-LXRα-ABCA1. Blocking HMGB1 release by EP or inhibiting TLR4 activation by TAK-242 almost reversed CUMS-induced the drownregulation of PPARγ-LXRα-ABCA1 and impeded the development of atherosclerosis in apoE^-/-^ mice. These results provide a novel insight into the mechanism of CUMS-induced atherosclerosis and offered HMGB1/TLR4 signaling could be a potential target for the prevention and treatment of this disease.

## Data Availability

All datasets generated for this study are included in the manuscript and/or the supplementary files.

## Author Contributions

H-FG contributed conception, design of the study, and drafted the manuscript. H-FG, NL, Z-QX, LH, HL, R-JZ, R-MC, and X-LZ performed the experiments. H-FG and NL analyzed the data for the work. D-FL and Y-LT critically revised the manuscript.

## Conflict of Interest Statement

The authors declare that the research was conducted in the absence of any commercial or financial relationships that could be construed as a potential conflict of interest.
